# Real-time approach to evaluate spinal cord topography of motor pool activation during gait

**DOI:** 10.3389/fbioe.2026.1753344

**Published:** 2026-07-21

**Authors:** Priscilla Avaltroni, Germana Cappellini, Francesca Sylos-Labini, Barbara La Scaleia, Francesco Lacquaniti, Yury Ivanenko

**Affiliations:** 1 Laboratory of Neuromotor Physiology, LIFE - Istituto di Ricerca e Cura - Santa Lucia IRCCS, Rome, Italy; 2 Department of Systems Medicine and Center of Space Biomedicine, University of Rome Tor Vergata, Rome, Italy; 3 Department of Systems Medicine, University of Rome Tor Vergata, Rome, Italy

**Keywords:** cerebral palsy, feedback, real-time gait assessment, rehabilitation, spinal maps

## Abstract

Real-time assessment of spinal motor output (“spinal maps”) can provide insight into how neural control strategies during walking differ between healthy individuals and patients with neurological disorders. Abnormal spatiotemporal activation of spinal motor pools may underlie gait impairments, making spinal maps a potential physiological marker for guiding personalized rehabilitation. We present a stride-by-stride online visualization method for estimating spinal locomotor output from multi-muscle EMG signals mapped onto the approximate rostrocaudal location of motor pools, identifying activation patterns across lumbar and sacral motor pools. The primary objective of this framework is to serve as a near real-time neurophysiological monitoring and assessment tool during clinical gait analysis. Beyond this core diagnostic capability, the system is designed as an extensible platform that may support real-time feedback to therapists, exploratory biofeedback to patients, or future integration into closed-loop spinal neuromodulation protocols. The system enables online visualization of spinal maps and related parameters (such as timing, intensity, and coactivation of lumbar and sacral motor pools) through a flexible, user-friendly software interface. Preliminary tests in eight children with cerebral palsy and comparing with typically developing peers show that, despite stride-to-stride variability, the method may effectively differentiate in real-time impaired from typical spinal activation patterns, supporting its promise for clinical gait rehabilitation.

## 1 Introduction

In recent decades, real-time gait assessment has gained substantial interest due to its ability to provide objective data in clinical settings and to automate the analysis of complex biomechanical information collected from wearable sensors, video systems, and other modalities. These developments offer a paradigm shift in clinical gait evaluation by enabling real-time capture and interpretation of gait parameters (Yang et al., 2011; Allseits et al., 2017; Abdelmohsen, 2025). Existing approaches focus primarily on kinematic and kinetic measurements, such as walking speed, distance travelled, joint angular ranges, joint torques, centre-of-mass motion, foot clearance, and movement symmetry (Massaad et al., 2010; Capogrosso et al., 2018; Martini et al., 2020; Nagano et al., 2020; Dusane et al., 2023; Zhao et al., 2024; Vacheva and Drumev, 2025). While these variables offer valuable general descriptors of gait performance, they allow only limited insight into the underlying neural control strategies or impairments in locomotor pattern generation.

Understanding spinal cord function is particularly critical for gait analysis because the executive components of the locomotor pattern-generating circuitry, including the final common pathway represented by spinal motoneurons (MN), reside in the spinal cord (Grillner and El Manira, 2015; Avaltroni et al., 2024). Neurological conditions such as cerebral palsy, spinal cord injury, spastic paraplegia, etc., can disrupt spinal circuitry integrity (Grasso et al., 2004; Cappellini et al., 2016; Martino et al., 2018) and result in substantial motor impairments. In addition, spinal maps have revealed distinct loci of motor pool activity across developmental and ageing stages - from early infancy (Ivanenko et al., 2013) to older adulthood (Monaco et al., 2010) - highlighting the importance of considering age-related changes in interpreting spinal locomotor output (Avaltroni et al., 2024).

In this context, our goal was to extend existing real-time approaches by estimating the global characteristics of spinal motoneuronal output during gait based on muscle activity patterns. Although recent neuroimaging advances have begun to illuminate spinal circuitry and MN recruitment (Stroman et al., 2014; Kinany et al., 2023; Nierula et al., 2024; Spedden et al., 2024), spinal fMRI remains technically limited - especially during dynamic tasks - due to motion artefacts, sensory contamination, and the inability to assess spinal activation in real time. In this study that specifically aimed at assessing the spinal locomotor output in real time, we used an approach originally developed in cats (Yakovenko et al., 2002) and later adapted for humans (Grasso et al., 2004; Ivanenko et al., 2006; La Scaleia et al., 2014; Wenger et al., 2016). This new approach consists in the simultaneous recording of kinematic and surface electromyographic (EMG) activity of many muscles and the subsequent schematic representation (map) of the activity of these muscles through the rostrocaudal localization of the corresponding MNs activated in the spinal cord. Importantly, these maps should not be interpreted as direct measurements of spinal neural activity, but rather as approximations derived from peripheral recordings and anatomical assumptions. The proposed spinal mapping approach assumes that EMG activity reflects the level of activation of the underlying motor units, and that each muscle can be associated with one or more spinal segments according to standardized myotomal innervation charts. By projecting muscle activity onto these segments, the resulting spinal maps provide an indirect, model-based representation of the distribution of motor output across the spinal cord rather than a direct measurement of spinal neural activity.

Our primary focus was placed on developing and technically validating a robust, real-time monitoring framework that provides an accessible, non-invasive assessment of the segmental organization of motor output during gait. By integrating Vicon motion-capture data with continuously streamed muscle activity recordings, this software acts as a clinical assessment platform capable of capturing individual, segment-specific adaptations stride-by-stride. While the platform architecture holds significant potential for guiding therapist interventions, driving visual biofeedback, or pairing with spinal stimulation strategies, these clinical extensions are framed as prospective future directions, with this paper serving as the essential proof-of-concept and feasibility validation. To our knowledge, no prior study has comprehensively assessed spinal motoneuron activation maps during gait in real time. Testing of the system’s performance - from stride detection to muscle activation processing and spinal map generation - confirmed reliable real-time functionality in a sample of children with cerebral palsy (CP) and typically developing (TD) children. In the context of rehabilitation, having a comprehensive overview of spinal activation may be more informative and effective than examining individual muscle activities alone. The proposed approach is not intended to replace standard clinical assessment but to complement it by providing insight into the segmental organization of motor output, which is not directly accessible through conventional gait analysis. Preliminary results indicate that the proposed approach can detect differences in spinal motor output between neurologically impaired and TD children. These findings underscore the potential of this method for investigating spinal motor control in real time and for supporting the assessment of locomotor function in paediatric populations with neurological conditions.

## 2 Materials and methods

### 2.1 Experimental setup and measurements

The experimental set-up can be used for both overground and treadmill walking, depending on the specific experimental or clinical application. In the present study, however, all participants performed overground walking trials only, to ensure consistency across subjects and avoid potential variability related to locomotion modality.

The proposed system is currently capable of processing consecutive strides from a single body side. This design choice was made to ensure robust real-time performance and minimal computational latency during online processing. Although this represents a limitation for the assessment of bilateral gait asymmetries, particularly relevant in clinical populations such as children with cerebral palsy, the system architecture can be extended to simultaneous bilateral analysis as it may provide additional insights into inter-limb coordination and asymmetry.

**FIGURE 1:**
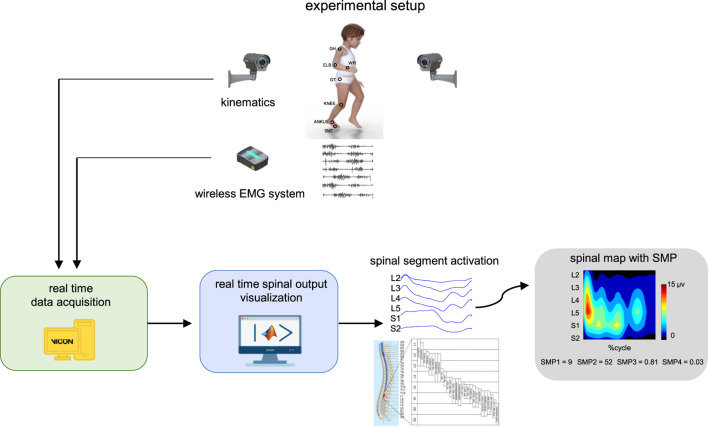
Real-time approach to evaluate spinal cord topography of MN activation during gait. Schematic representation of the experimental setup. While the child walks, kinematic and EMG data are streamed from Vicon-Nexus to a second computer for stride-by-stride online visualization of spinal spatiotemporal maps. On the bottom right, innervation of lower limb muscles is schematically depicted (from Sharrard, 1964). At the end of each stride, the total motor output pattern of any given (L2–S2) spinal segment is reconstructed and then the spinal map and the associated spinal map parameters (SMPs) are displayed on the screen.

For real-time evaluation of the spinal motor pool activation within the gait cycle, both lower limb kinematics and multi-muscle EMG signals are recorded (Figure 1). Kinematics of one single body-side, right or left side, was recorded at 200 Hz by means of Vicon-Nexus system with 10 cameras placed around the walking path. Infrared reflective markers are attached on each side of the child to the skin overlying the following landmarks: gleno-humeral joint (GH), elbow (ELB), wrist (WR), greater trochanter (GT), lateral femur epicondyle (KNEE), ankle (ANKLE) and the fifth metatarsophalangeal joint of the foot (5 MT). Although the primary focus of the study is lower-limb locomotion, upper-body markers were included to ensure a complete reconstruction of whole-body kinematics and to support potential future analyses of postural control and intersegmental coordination. Kinematic data were used in this study primarily for gait-cycle segmentation and temporal normalization of EMG signals. Specifically, they allow the identification of stride phases, enabling the alignment of muscle activity across strides, and general gait parameters (walking speed and stride length). Kinematic input does not directly contribute to the spinal mapping itself, but is essential for ensuring consistent temporal analysis of the EMG signals.

While wearable inertial measurement units (IMUs) or markerless computer vision systems offer clear logistical advantages for widespread, lower-cost deployment in standard clinical workflows, an optoelectronic multi-camera arrangement was utilized in this validation study to provide a high-precision, sub-millimetre reference baseline. This level of accuracy was critical during the development phase to ensure that no positional tracking errors or sensor artefacts contaminated the online leg elevation angle calculation or the subsequent temporal normalization of the spinal maps. Crucially, the custom data streaming and MATLAB processing pipeline is architecturally hardware-agnostic. Because communication is handled via generic commanded streams, the algorithmic framework can be directly integrated with streaming outputs from single-camera markerless systems or real-time IMU arrays, supporting broad clinical generalizability.

EMG activity was recorded from a single body side by means of surface electrodes from the muscles that contribute mostly to the overall spinal maps and also constitute a relatively large part of the total cross-sectional area of lower limb muscles: long head of biceps femoris (BF), semitendinosus (ST), rectus femoris (RF), vastus lateralis (VL), vastus medialis (VM), gastrocnemius medialis (MG), gastrocnemius lateralis (LG), soleus (SOL), tibialis anterior (TA). All EMGs were recorded at 2000 Hz using the Trigno Wireless EMG System (Delsys Inc., Boston, MA), bandwidth of 20–450 Hz, overall gain of 1000, synchronized with the Vicon-Nexus system. Although a limited set of muscles was recorded, previous studies have shown that these muscles contribute most strongly to the reconstruction of locomotor spinal maps and their segmental organization (Ivanenko et al., 2013; La Scaleia et al., 2014). Specifically, they represent the major flexor and extensor muscle groups of the thigh and shank, thereby capturing the principal features of motor pool activation within the lumbosacral spinal cord. Furthermore, these muscles account for a substantial proportion of the total lower-limb muscle cross-sectional area (Ward et al., 2009), making them suitable for characterizing the overall locomotor spinal output. The system can accommodate up to 16 EMG channels, allowing the inclusion of additional muscles such as gluteus maximus, gluteus medius, iliopsoas, tensor fasciae latae, sartorius, peroneus longus, and flexor digitorum brevis. However, some of these muscles (e.g., iliopsoas and flexor digitorum brevis) are more prone to recording artefacts and require additional signal-quality verification. In the present proof-of-concept study, we therefore focused on the subset of muscles previously demonstrated to provide the most informative representation of spinal motor output while minimizing computational demands and ensuring robust real-time processing.

At the start of the recorded session, a static calibration trial was conducted using the Vicon- Nexus system. This trial provided the basis for automatic labelling of markers during subsequent walking trials. It was essential that all markers were visible and correctly tracked during the static trial to ensure accurate and reliable labelling.

### 2.2 Data streaming and data pre-processing

To enable stride-by-stride online visualization of spinal spatiotemporal maps of motor pool activation during gait, a custom data acquisition and processing framework was developed. This system was designed to provide immediate computation and display of spinal maps and related parameters during walking, using kinematic and electromyographic data streams in real-time. The resulting real-time evaluation system streams data from the Vicon-Nexus software, which communicates with the Trigno Wireless EMG System, directly into Matlab (MathWorks Inc., Natick, MA, USA), and is supported by a two-computer architecture in which one machine runs the Vicon DataStream Server together with the Trigno Wireless EMG System, while the second runs Matlab (Figure 1). Communication between Vicon-Nexus and Matlab is implemented via a dedicated TCP/IP connection using the Vicon DataStream SDK and API Dynamic Link Library, allowing high-level commands to be executed seamlessly from within Matlab. Specifically, Vicon DataStream SDK version 1.11 was employed to transmit real-time data from Vicon-Nexus to Matlab 2024a, where custom scripts process the incoming stream, encompassing both kinematic data from each marker and EMG data from all recorded muscles. Dedicated Matlab routines were developed to extract, organize, and analyse these data from the stream transmitted by Vicon-Nexus via the SDK protocol, leveraging Matlab’s extensive scientific, graphical, and customization capabilities to support real-time evaluation and visualization.

**FIGURE 2:**
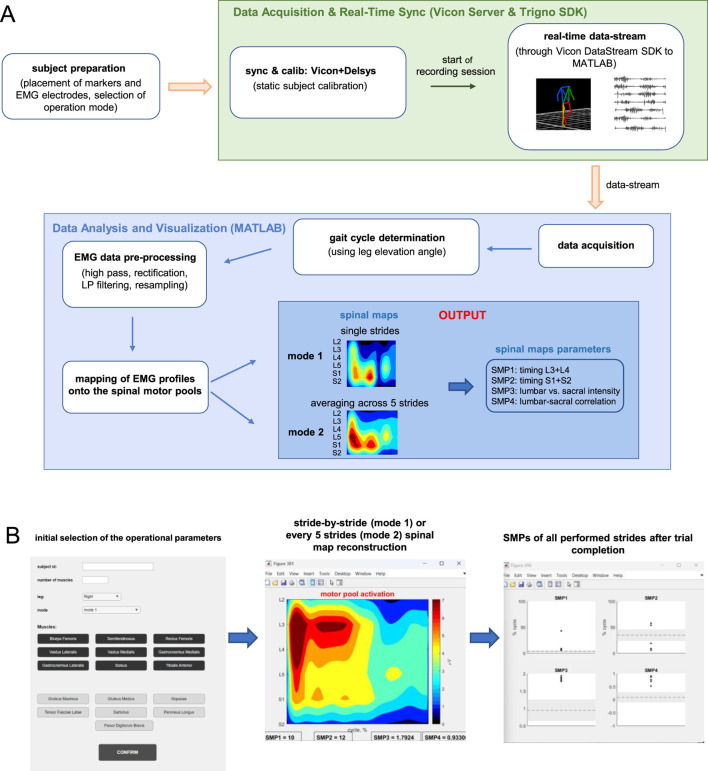
Flow diagram of the near real-time data processing pipeline and software user interface layout. **(A)** The steps in the flow diagram include real-time acquisition of kinematic and EMG data, gait cycle recognition, pre-processing of the data in Matlab, reconstruction of the spinal motor pool activation, and visualization of the spinal map and corresponding SMPs. **(B)** The graphical user interface windows demonstrate: the initial configuration of operational parameters and body-side target channels (left panel); the near real-time visualization of segment activation and running parameters rendered after cycle (mode 1) or five cycles (mode 2) termination (middle panel); and the aggregate plotting of accumulated metrics following trial completion (right panel), where individual circles denote separate strides and the underlying horizontal grey bands define the normative baseline (mean ± SD) calculated from reference TD children.

Prior to mapping the EMG signals onto the lumbosacral motor pools, the incoming hardware-synchronized stream is evaluated sequentially in MATLAB to coordinate gait cycle recognition and signal pre-processing (Figure 2A). Frame-by-frame kinematic data are continuously processed to determine gait events using the maximum value of the limb elevation angle (Dominici et al., 2007), calculated from the GT to the 5 MT marker. The software monitors this leg oscillation profile to identify stride onset and termination in real time. Only after the completion of a full gait cycle is confirmed by this kinematic routine does the software analyse the corresponding multi-muscle EMG data recorded over that specific cycle and initiate the pre-processing pipeline (Figure 2A). This triggered EMG data pre-processing sequence includes: high-pass filtering (by default 60 Hz), full-wave rectification, low-pass filtering with a zero-lag fourth-order Butterworth filter (by default 5 Hz) to obtain envelope time series, and immediate resampling via time interpolation over a normalized n-point time base (by default 200 points) to prepare the cycle data for topographic reconstruction. These pre-processing parameters were selected based on established procedures previously used in studies of spinal motor output during gait in both TD children and children with cerebral palsy (Sylos-Labini et al., 2022; Villani et al., 2024; Avaltroni et al., 2026), thereby ensuring methodological consistency and comparability across datasets. The 60-Hz high-pass filter was used to minimize low-frequency movement artefacts and baseline fluctuations that are common during walking, while the subsequent low-pass filtering at 5 Hz was intended to extract the smooth EMG envelope underlying the general spatiotemporal pattern of motor pool activation. Because the present study focused on the reconstruction of single-stride spinal maps rather than detailed analyses of rapid muscle activation dynamics, a relatively low cut-off frequency was selected to emphasize the overall activation profile across the gait cycle. Interpolation was used for temporal normalization of the gait cycle and to facilitate comparison across strides. Although these parameter values may not be optimal for all populations or applications, they have been successfully adopted in previous spinal-mapping studies and provide a robust framework for the present proof-of-concept implementation.

### 2.3 Spinal maps reconstruction

Once the EMG signals have been rectified and filtered and the gait cycle has been identified, the software employs a dedicated algorithm to map these signals onto the rostrocaudal anatomical position of MNs within the spinal cord. To reconstruct and characterize the spatiotemporal organization of the total motor output pattern of any given (L2–S2) spinal segment, the recorded profiles of EMG activity were mapped onto the rostrocaudal location of MN pools in the human spinal cord using the myotomal charts of Kendall et al. (2005). The method assumes that the EMG amplitude is related to the level of activation of the corresponding motoneuron pools and the myotomal distribution of muscles is consistent across individuals. The resulting spinal maps should be interpreted as a model-based approximation of segmental motor output, derived from peripheral muscle activity and anatomical assumptions. Although indirect, this approach is supported by evidence that rectified EMG amplitude provides a reasonable estimate of the aggregate activity of the underlying motor-unit population and has been successfully used in previous studies to characterize locomotor spinal output (Ivanenko et al., 2006; Monaco et al., 2010) and to guide epidural stimulation in spinal cord injured patients (Rowald et al., 2022). This approach provides an interpretation of the motor pool activation at a segmental level rather than at the individual muscle level (Yakovenko et al., 2002; Grasso et al., 2004; Ivanenko et al., 2008; Monaco et al., 2010; Wenger et al., 2016) and it can be used to characterize the spinal locomotor output by considering relative intensities, spatial extent, and temporal structure. In general, each muscle is innervated by several spinal segments, and each segment supplies several muscles. To reconstruct the motor pool output pattern of any given spinal segment *S*_*j*_ of the lumbosacral enlargement (L2-S2) most active during locomotion, all rectified EMG-waveforms corresponding to that segment were averaged using appropriate weighting coefficients according to the following equation:

$$S_{j}\left(t\right)=\frac{\sum_{i=1}^{n_{j}}k_{ij.}EMG_{i\left(t\right)}}{n_{j}}$$ (1)

where nj is the number of $EMG_{i}$ waveforms (expressed in µV) corresponding to the $j_{th}$ segment and $k_{ij}$ is the weighting coefficient for the $i_{th}$ muscle (X and x in Kendall’s chart were weighted with $k_{ij}=1$ and $k_{ij}=0.5$ , respectively). The weighting coefficients were derived from Kendall’s myotomal charts, which specify the spinal segments associated with each muscle. In these charts, a capital “X” indicates a primary segmental innervation, whereas a lowercase “x” indicates a secondary contribution. Accordingly, higher weighting coefficients were assigned to primary innervation segments and lower coefficients to secondary segments. For each spinal segment, the estimated motor output was computed as the sum of the rectified EMG activities of all recorded muscles weighted according to their segmental innervation pattern. The resulting segmental activity estimate (Sj) was then scaled according to the relative number of motoneurons present at each spinal level, normalized to L3, which contains the largest motoneuron population (Tomlinson and Irving, 1977). This normalization primarily affects the boundary segments (e.g., L2 and S2), which contain substantially fewer motoneurons than the central segments of the lumbosacral enlargement (for instance, for L2, we multiplied *S*_*j*_ by 0.4, and, for S3, we multiplied it by 0.33 in order to better account for the heterogeneity in the MN pools along the lumbosacral enlargement). Thus, the weighting coefficients and normalization factors represent anatomical constraints derived from established myotomal organization and segment-specific motoneuron distributions. The resulting spinal maps should therefore be interpreted as indirect, model-based estimates of segmental motor output rather than direct measurements of spinal neural activity. To visualize a continuous smoothed rostrocaudal spatiotemporal activation of the spinal cord, we used a filled contour plot that computes isolines calculated from the activation waveform matrix and fills the areas between the isolines using separate colours.

The spinal map and its associated parameters, which will be described in detail in the following section, are displayed after each stride in real-time on the screen, providing immediate visual feedback on the organization of spinal motor output during each stride.

### 2.4 Quantitative metrics for clinical assessment of spinal maps (SMP1–SMP4)

In order to evaluate the general spatiotemporal features of the lumbosacral MN output during gait, the following spinal map parameters (SMPs) are chosen as performance indicators to characterize the spinal maps of MN activity, based on their helpfulness to distinguish between normal and atypical motor pool activity during walking reported in our previous studies.

- SMP1: timing of the maximal activation of the dominant lumbar (sum of activity from L3 and L4, L3-L4) spinal segments throughout the gait cycle (reported in % of gait cycle);
- SMP2: timing of the maximal activation of sacral (sum of activity from S1 and S2, S1-S2) spinal segments throughout the gait cycle (reported in % of gait cycle);
- SMP3: the ratio between the mean motoneuron activity in the lumbar L3-L4 and sacral S1-S2 segments.
- SMP4: similarity of lumbar and sacral segments calculated as correlation coefficient between the mean motoneuron activity in the lumbar L3-L4 and sacral S1-S2 segments.

We chose these parameters because they capture complementary and physiologically meaningful aspects of segmental motor control during gait, including timing, duration, complexity, and regularity of activation. Together, SMP1-SMP4 provide a concise yet comprehensive description of spinal motor output dynamics, allowing for the characterization of both typical and pathological gait patterns. Furthermore, these parameters have been previously shown in our studies to differentiate between TD children and children with cerebral palsy, supporting their sensitivity and potential clinical relevance (Ivanenko et al., 2013; Cappellini et al., 2016; Avaltroni et al., 2026).

To guide clinical interpretation during real-time monitoring, the physiological relevance and manifestation of typical versus pathological values for each parameter are defined as follows.

- SMP1 and SMP2 (segmental timing): In typical gait, these values demonstrate a pronounced temporal segregation. Typical values for SMP1 (lumbar peak) occur at early stance (heel strike/loading response) to control weight acceptance, while SMP2 (sacral peak) shifts to late stance to drive terminal push-off (Ivanenko et al., 2013). Pathologically, children with CP often demonstrate near-identical or merged values for SMP1 and SMP2 (Avaltroni et al., 2026). This lack of differentiation alerts the therapist to a loss of selective motor control and a primitive, synchronous spinal drive.
- SMP3 (lumbar-to-sacral intensity ratio): This metric quantifies the relative distribution of neural drive across the lumbosacral enlargement. A typical baseline value balances proximal and distal demands. An abnormally elevated SMP3 indicates hyper-intensity of the lumbar motor pools. This guides the clinician to identify specific proximal compensations, such as quadriceps hyperactivity or loading adjustments related to a crouch gait and a flexed knee posture during stance.
- SMP4 (waveform similarity and coactivation index): This parameter provides a direct measure of coactivation between distant spinal segments. In a typical neuro-intact system, SMP4 values are low and not significantly different from zero, indicating highly independent, specialized modulation of the lumbar and sacral outputs. Conversely, elevated, positive SMP4 values indicate high waveform similarity, providing the therapist with an immediate, objective marker of pathological multi-segment coactivation and central hyperreflexia.

These SMPs are displayed in real-time stride-by-stride on the screen alongside the corresponding spinal map for each individual stride, providing immediate feedback on the organization of spinal motor output. Simultaneously, the software records these parameters for every stride, enabling *post hoc* analysis. At the end of the walking trial, the operator can review the mean values of each parameter across all steps, examine inter-stride variability, and perform statistical analyses to quantify differences in the temporal and spatial characteristics of spinal motor activity, providing a summary of spinal motor output across the entire walking sequence. This combined real-time visualization and cumulative analysis allows for a comprehensive assessment of locomotor control, highlighting both transient and consistent features of spinal activation patterns within and across participants.

### 2.5 Modes of operation and user interface

The software operates in two distinct modes (Figure 2A), providing flexibility in the analysis of spinal locomotor output. In the first mode, the system generates and displays the spinal map and its associated parameters after each stride. This mode allows for stride-by-stride visualization, enabling real-time observation of intra-stride variability and immediate feedback. In the second mode, the software computes the spinal map based on the average EMG signals across a user-defined number of strides (*n*). Once the *n* strides have been completed, the averaged spinal map and its corresponding spinal parameters are displayed. This approach is particularly useful for reducing stride-to-stride variability and obtaining a more stable representation of the subject’s overall idiosyncratic spinal activation pattern. Together, these two modes offer complementary insights: one captures dynamic changes across strides (with an option of potential biofeedback to patients to adapt their pattern stride-by-stride), while the other emphasizes consistency and mean behaviour over multiple gait cycles.

Processing methods are customizable, allowing users to adjust parameters (filter cutoff frequencies, the number of interpolation points, selected muscles, mode of operation) and analysis methods (e.g., Sharrard or Kendall). For the latter choice, while the default method is that based on the Kendall charts, the operator can also use the Sharrard (1964) data table for innervation (Ivanenko et al., 2008; Martino et al., 2018), which approximates the proportion of total muscle activation attributable to each segment (by taking multiple slices within each spinal segment), instead of assuming equal proportions in all segments. To this end, we subdivided each segment into six subsegments and applied the same Equation 1 for each j_th_ subsegment (Ivanenko et al., 2006). The resulting spinal cord maps of activation were not smoothed as in the case of the Kendall chart but instead they contained 36 discrete bands for visualization. Still, for the SMP performance indicators, the same procedure is used (by summing up the slices belonging to the same spinal segment resulting in six lumbosacral segments from L2 to S2). Finally, the advanced user has the flexibility to modify the script as desired (for instance, including the spinal maps of the cervical segments for the locomotor tasks involving prominent activity of the upper limbs), allowing for the integration of custom algorithms to extend or adapt the analysis according to specific research needs.

### 2.6 Testing of software for the evaluation of spinal cord locomotor output in children with CP and TD children

To test the system, we selected eight children with a clinical diagnosis of CP who were able to walk independently, recruited from the Department of Pediatric Neurorehabilitation of IRCCS Santa Lucia Foundation, and two TD children. The characteristics of children with CP and walking trials are shown in Table 1. The Ethics Committee of IRCCS Santa Lucia Foundation approved the study procedures (protocol n. CE/PROG.875) that adhered to the Declaration of Helsinki for medical research involving human participants, and informed written consent was obtained from the parents of all children. Children with CP were diagnosed based on clinical history, neurological examination, and brain MRI findings. Motor type classification followed the Surveillance of Cerebral Palsy in Europe (SCPE) criteria (Himmelmann et al., 2017), distinguishing unilateral and bilateral spastic forms, as well as dyskinetic and ataxic presentations where applicable. Inclusion criteria included independent walking ability and a Gross Motor Function Classification System (GMFCS) levels I-II. Ankle plantarflexor spasticity was assessed using the Modified Ashworth Scale (MAS) (Bohannon and Smith, 1987). Exclusion criteria included orthopaedic surgery within the previous year and botulinum toxin injections within the previous 4 months. All children were able to walk independently during the experimental sessions. If children used an ankle-foot orthosis (AFO) in daily life, it was removed for the duration of the experiment.

**TABLE 1:** Characteristics of children with CP. The speed (V, m/s) is the average speed over all strides. The stride length is normalized to limb length (LL, estimated as thigh + shank segments).

Subjects	Gender	GA, wk	BW, gr	Age, yrs	Body height, m	Body mass, kg	TCP, SCPE	MAS (L/R)	GMFCS	Lesion type	# Trials	Strides, n	V, m/s	Stride length, LL
CP1	M	30	1560	3	0.62	14	Spastic bilateral	1/2	1	PWM	3	20	0.54 ± 0.09	1.28 ± 0.15
CP2	M	36	2500	3	0.73	15	Ataxic	0/0	1	PWM	3	20	0.58 ± 0.08	1.26 ± 0.13
CP3	M	25	940	3.5	0.71	15.5	Ataxic	0/0	1	PWM	3	20	0.30 ± 0.15	0.89 ± 0.11
CP4	F	26	900	4	0.64	14	Spastic bilateral	0/0	2	PWM	3	20	0.47 ± 0.09	1.32 ± 0.10
CP5	F	40	3510	4	0.80	15.5	Spastic monolateral	3/0	2	PWM	3	20	0.58 ± 0.08	1.34 ± 0.12
CP6	F	27	860	5	0.87	18	Spastic monolateral	3/0	1	PWM	3	20	0.88 ± 0.05	1.45 ± 0.14
CP7	M	25	750	7	1.03	24	Spastic monolateral	2/0	2	PWM	3	20	0.72 ± 0.04	1.62 ± 0.21
CP8	M	31	1900	12	1.21	40	Spastic bilateral	1/2	1	PWM	3	15	0.55 ± 0.07	1.03 ± 0.15

Abbreviations: GA, gestational age at birth; BW, birth weight; SCPE, surveillance of cerebral palsy in europe; MAS, Modified Ashworth Scale (the rater graded each ankle plantarflexion spasticity: L-left leg, R-right leg); GMFCS, gross motor function classification system; PWM, periventricular white matter lesions; stride number refers to the total number of strides recorded and analysed in three walking trials.

**TABLE 2:** Mean counts of limb MNs in the individual segments of the human spinal cord (13–40 years, 12 cases).

Segment level	
L1	806
L2	5146
L3	12765
L4	12069
L5	12674
S1	10372
S2	4216
S3	409
Total number	58457

Data are adopted from the study by Tomlinson and Irving (1977).

The TD cohort consisted of 26 children (12 males, 14 females; age range: 3–12 years). Two children were tested using the real-time system, while data from additional 24 TD children, previously acquired and processed offline (Cappellini et al., 2016), were included to establish an age-matched reference dataset. Across all TD participants, the mean number of analysed strides was 71 ± 25 and the mean walking speed was 0.82 ± 0.15 m/s.

Experiments were performed in the Laboratory of Neuromotor Physiology, IRCCS Santa Lucia Foundation, by the gait analysis experts in the presence of a neuropaediatrician, a physiotherapist, and one or both parents of the child. All children were evaluated during three walking trials on the floor for 8 m in a straight path at a preferred self-selected speed in a 11 m × 14 m laboratory room. Prior to data recording, we performed static subject calibration to verify the marker visibility needed for online kinematic body motion reconstruction, and checked the quality of EMG signals by asking the subjects to perform contractions (lack of artefacts, noise, appropriate placement of electrodes). The defaults parameters were used for real-time assessing the spinal maps of MN activation (operation mode 1, Kendall charts of muscle innervation, 9 recorded muscles of the right leg, and default EMG pre-processing values). During walking, the operator visualized and monitored the spinal maps and their relative SMPs.

Since the SMPs were recorded for all performed strides, we examined offline for each participant the significant differences. The differences between SMP1 and SMP2 were evaluated using circular statistics (Watson–Williams test) (Berens, 2009). Statistics on correlation coefficients (SMP4) was performed on Z-transformed values to ensure normality, and we evaluated whether SMP4 was significantly different from zero using a one-sample, one-tailed t-test. Low correlation values indicate low similarity in lumbar (L3+L4) and sacral (S1+S2) motor pool activation, which is typical for normal gait since lumbar and sacral activities follow different time courses (Cappellini et al., 2016; Villani et al., 2024). Conversely, high correlation values (significantly greater than zero) may indicate a pathological coactivation of these segments, where a higher SMP4 value reflects a greater degree of lumbosacral coactivation. Reported results are considered significant for p < 0.05.

## 3 Results

### 3.1 System latency and near real-time software performance

To characterize system performance, all real-time metrics were computed stride-by-stride during walking in a representative subject to obtain estimates of system timing and assess its behaviour under realistic operating conditions. The processing latency was quantified using MATLAB tic-toc functions, measuring the time required for stride detection, EMG processing, spinal map reconstruction, and computation of SMPs. The resulting processing latency was ∼10 m. The visualization latency was also estimated in MATLAB using the tic-toc function applied immediately before and after the plotting routine, corresponding to graphical rendering and GUI updates. This component was ∼50 m and represented the substantial contribution to the overall system delay.

The end-to-end latency, defined as the time elapsed between a biomechanical event (heel strike) and the corresponding visualization of the updated spinal map, was assessed using an external synchronization approach. An accelerometer placed on the ankle was used to detect heel strike events, identified as the acceleration burst occurring at foot-ground contact. This signal was synchronized with a photodiode-based system that detected a flickering square displayed on the graphical interface each time a new spinal map was plotted (alternating black/white state at each update). This setup allowed precise temporal alignment between the physiological event and the visual output. Using this method, the end-to-end latency was estimated to be ∼80 m. This latency also includes contributions related to data streaming and acquisition, which depend on the motion-capture system and on real-time marker labelling and tracking procedures. EMG and kinematic signals were inherently synchronized at the acquisition level through the Vicon DataStream SDK, which streams both modalities within a unified hardware-synchronized framework, thereby eliminating the need for *post hoc* temporal alignment. If necessary, this end-to-end latency can be further reduced in future developments using other programming languages (e.g., Python, C++, Fortran) or a more powerful PC with faster visual rendering capabilities. Nevertheless, given that the typical stride duration in patients is > 1 s, the reported end-to-end latency is well within an acceptable threshold for the near real-time analysis of spinal maps.

To further evaluate metric robustness and system consistency, SMP1-SMP4 parameters were compared between real-time and offline analyses of the same strides in a representative subject: SMP1: 16.1 ± 10.1 %cycle (real-time) vs. 17.4 ± 10.7 %cycle (offline), SMP2: 26.1 ± 18.3 vs. 24.4 ± 12.5, SMP3: 1.74 ± 0.09 vs. 1.78 ± 0.08, and SMP4: 0.75 ± 0.80 vs. 0.84 ± 0.94. Overall, real-time and offline values showed highly comparable trends, supporting the robustness and reliability of the real-time implementation.

### 3.2 General gait characteristics and experimental demographics

All children completed three straight-path walking trials without assistance. The TD reference dataset comprised 26 children (12 males, 14 females; age range 3–12 years), with a mean of 71 ± 25 analysed strides per participant and a mean walking speed of 0.82 ± 0.15 m/s. For the CP cohort, 15–20 strides per participant were available for analysis (Table 1). Walking speed ranged from 0.30 to 0.88 m/s in children with CP and from 0.56 to 0.98 m/s in TD children. Normalized stride length in the CP cohort ranged from 1.03 ± 0.15 to 1.62 ± 0.21 limb lengths. Detailed demographic, clinical, and gait characteristics are reported in Table 1.

### 3.3 Stride-by-stride online reconstruction of spinal topography

Using the spinal motor imaging technique, we examined changes in spinal maps and associated parameters based on EMG activity and corresponding segmental innervation during gait. The stride-by-stride online visualization of spinal spatiotemporal maps reliably captured the dynamic rostrocaudal activation of lumbar and sacral motor pools at each stride. Once the end of the gait cycle is detected, the software immediately computes the subject-specific spinal map. The observed delay between gait-cycle termination and map visualization is therefore due exclusively to MATLAB’s internal graphical rendering processes and to display refresh frequency time (within the frame rate of the monitor).

**FIGURE 3:**
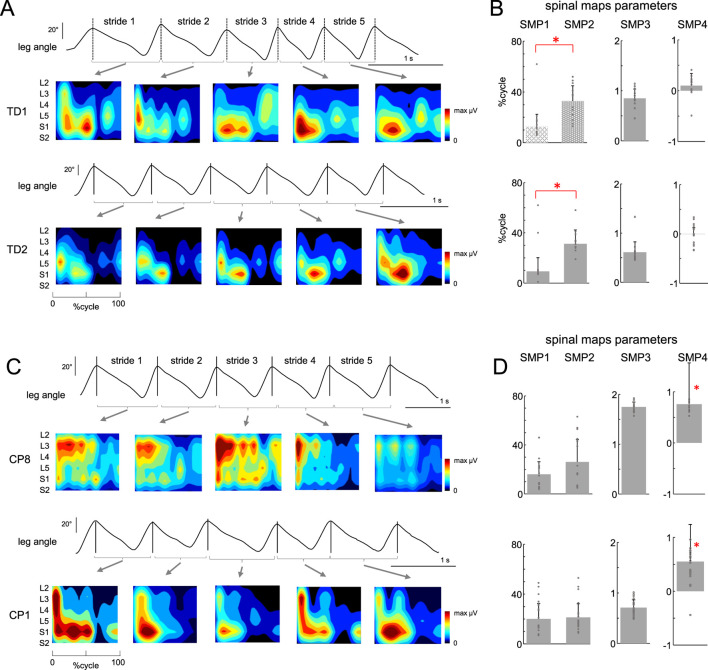
Usage of the software for the evaluation of spinal cord topography in TD children and in children with CP. **(A)** Examples of the spinal maps during five consecutive strides in two TD children. Upper panel: leg oscillation angle, vertical lines correspond to the start and end of each stride (can be visualized at the end of the walking trial). Lower panel: spatiotemporal unilateral maps of MN activity as a function of gait cycle and spinal segment level (L2−S2) reconstructed using the EMG envelopes of the ipsilateral leg and location of MN-pools in the human spinal cord. Every spinal map is plotted in real-time after each stride (Mode 1, Figure 2A). To better depict the relative intensities of lumbar and sacral motor pool activation, a colour scale (in µV, not shown) was maximized for each stride. **(B)** Spinal map parameters (mean + SD) for all strides in all three trials performed by each child. Circles denote the distribution of values in individual strides. Asterisks and horizontal lines denote significant differences between SMP1 and SMP2 (circular statistics, Watson–Williams test, p < 0.05). Asterisks on SMP4 indicate that the Fisher z-transformed correlation between lumbar (L3–L4) and sacral (S1–S2) spinal segment activation waveforms is significantly greater than zero (one-tailed t-test, p < 0.05). **(C,D)** Spatiotemporal spinal maps and quantitative parameters plotted in an identical format for two representative children with CP (CP1 and CP8, Table 1). Note the lack of distinct temporal segregation between lumbar and sacral segments (SMP1 vs. SMP2) and the significant correlation (*) (one-tailed t-test, p < 0.05) indicative of pathological segmental coactivation (SMP4).

Examples of five consecutive strides from two TD children and two children with CP are shown in Figures 3A,C. The software automatically detected stride onset and termination in real time using the leg oscillation angle, which is plotted alongside the spinal maps. As expected, inter-stride variability was present, reflecting natural fluctuations in motor output. Nevertheless, consistent features of motor pool activation could be identified in both TD and CP children. Visual inspection of the maps allows the experimenter to determine whether lumbar and sacral loci of activation appear distinct—as in typical gait, with lumbar activation at early stance and sacral activation at late stance—or whether they are coactivated or follow atypical patterns.

In addition to visual assessment, the software computes four quantitative spinal map parameters (SMP1–SMP4) for each stride, which are both plotted and stored in real time. These parameters provide a concise summary of segmental activation timing, amplitude distribution, and waveform similarity. The saved parameters for all recorded strides in the four children are displayed in Figures 3B,D, illustrating clear differences between TD children and those with CP.

Finally, after the trial completion, we implemented the summary of the results for the performed strides by plotting on the screen the final graphs with values of SMPs for individual strides with respect to the normative reference range of TD children (Figure 2B). This operational mode calculates whether there is a statistical difference between SMP1 and SMP2, and whether SMP4 is significantly different from zero (indicative of pathological coactivation of lumbar and sacral segments; Figures 3B,D), so that the therapist or experimenter can completely assess the overall cumulative results after completing the block of strides.

### 3.4 Comparative evaluation of segmental activation patterns

In the reference TD children, spinal maps exhibited a pronounced separation of lumbar and sacral activation. Lumbar segments were most active during early stance, while sacral segments dominated late stance, consistent with the mature pattern observed in healthy adults (Ivanenko et al., 2013). To better depict the relative intensities of lumbar and sacral motor pool activation, a colour scale (in µV, not shown) was maximized for each stride. This rostrocaudal differentiation is reflected in the significant temporal difference between SMP1 and SMP2 (Figure 3B). Moreover, the low correlation between lumbar and sacral activation waveforms (SMP4) indicates largely independent modulation of these segmental outputs, supporting a well-organized, segment-specific motor control strategy.

**FIGURE 4:**
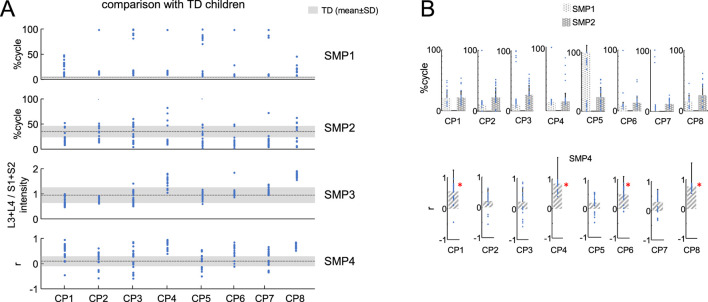
Spatiotemporal topographic map parameters across eight children with CP. **(A)** Aggregate summary metrics for all eight evaluated children with CP plotted against the normative baseline range of TD peers (grey shading, mean + SD). Circles display the distribution of values across all performed strides. **(B)** Statistical evaluation of segmental activation structure across children. The absence of asterisks over the temporal timing parameters (upper panel) indicate the absence of a statistically significant separation (Watson–Williams test, p > 0.05) between peak lumbar (SMP1) and sacral (SMP2) activation hotspots determined via circular statistics. Asterisks on the waveform similarity index (SMP4, lower panel) indicate that the mean Fisher z-transformed correlation coefficients between the lumbar (L3+L4) and sacral (S1+S2) segment waveforms are significantly greater than zero (one-tailed t-test, p < 0.05), confirming the presence of lumbosacral coactivation.

In contrast, children with CP showed a markedly different pattern. Figures 3C,D illustrates the results of the real-time analysis in two children with CP. Spinal maps revealed synchronous activation of lumbar (L3–L4) and sacral (S1–S2) segments, particularly around the onset of stance, consistent with the quasi-simultaneous activation previously described in many children with CP (Cappellini et al., 2016). This reduced rostrocaudal differentiation was reflected in similar values of SMP1 and SMP2 and in the high correlation of lumbar and sacral waveforms (Figures 3D, 4B). In addition, the CP8 child exhibited increased activation intensity in the lumbar motor pools (SMP3; Figure 2D), likely reflecting a slightly flexed knee posture during stance and the associated quadriceps activity, which is innervated by lumbar segments.

Figure 4 shows the results of the real-time evaluation in all recorded children with CP. Panel A shows the aggregate summary metrics plotted against the normative baseline range of TD peers, while panel B summarizes the statistical evaluation of the parameters: similar values of SMP1 and SMP2 in all eight children (Watson–Williams test, p > 0.05) and the relatively high (significantly different from zero, one-tailed t-test, p < 0.05) correlation of lumbar and sacral waveforms (SMP4), confirming the presence of lumbosacral coactivation. Together, these features indicate a more coupled and less segmentally specialized activation pattern, characteristic of altered spinal motor control in CP. An example of real-time processing for the CP8 child, including the same five strides shown in Figure 2C, is provided in Supplementary Video S1.

## 4 Discussion

In this study, we aimed to advance existing real-time gait assessment modalities by estimating global features of spinal motoneuronal output from multi-muscle peripheral activity patterns. To this end, we developed and tested a technical framework that integrates motion-capture data with continuously streamed EMG signals to generate near real-time, stride-by-stride visualizations of spinal motor pool activation during walking (Figures 1, 2). Comprehensive performance benchmarking demonstrated reliable functionality, minimal computational latency, and robust stride-detection across both TD children and peers with cerebral palsy (Figures 3, 4).

### 4.1 Clinical value: complementing the standard of care and prognosis

An important question in the evaluation of translational neurotechnologies is what unique value a new method adds compared to standard clinical care and traditional gait prognosis. Traditional gait analysis relies primarily on kinematic and kinetic variables - such as joint angles, torques, and spatiotemporal parameters - which offer essential behavioural descriptors of gait performance but provide limited direct insight into the underlying neural control strategies or structural deficits in locomotor pattern generation. The proposed spinal mapping approach is not intended to replace these established clinical metrics, but rather to complement them by providing an accessible window into the segmental organization of spinal motor output that is inherently obscured during conventional analysis.

The clinically complementary nature and prognostic potential of mapping segmental spinal drives are underscored by several key considerations.

- Capturing hidden neurodevelopmental biomarkers: Recent evidence demonstrates that crucial features of neuromuscular organization during development cannot be resolved by simpler methods or behavioural observation alone. In our recent large-scale cross-sectional study characterizing lower limb modularity (Avaltroni et al., 2026), we revealed that children with CP exhibit a profound lack of spinal locomotor maturation prior to the onset of independent walking. Specifically, these children conserve an immature, low-dimensional control structure (restricted to two basic activation modules) and present highly diffused, prolonged loci of motoneuron activity across the lumbosacral segments. Strikingly, the addition of new neuromuscular modules (fractionating into a mature four-module control strategy) and the localized differentiation of proximal versus distal extensor activity emerge selectively in correlation with the specific experience of independent walking. Conversely, children with severe, non-ambulatory CP retain an innate, newborn-like spinal activation topography regardless of chronological age. Because standard clinical scales often fail to differentiate underlying neural capacity from transient behavioural compensations, our real-time framework provides an indispensable tool for identifying these hidden neurophysiological markers of disease severity and tract integrity directly at the clinic baseline, especially during critical developmental windows for spinal pattern generation circuitry maturation (Avaltroni et al., 2026).
- Immediate, adaptive monitoring of therapeutic progress: Because this system visualizes the organization of spinal motor output on a stride-by-stride basis, it is uniquely suited for monitoring the immediate, acute progress of a patient during a session. Instead of relying on *post hoc* offline processing, clinicians can observe changes in lumbosacral activation dynamics synchronously with the task, capturing both transient variations and stable idiosyncratic adaptations over a sequence of steps. In this regard, one should notice that the images of motoneuron activation in the lumbosacral cord provide a synthetic view that is immediately understandable. The global picture of stride-by-stride activity can help the therapist to guide the patient toward a progressive adaption of their effort.
- Generalizability beyond paediatric populations: While validated here in paediatric cohorts, the underlying neurophysiological rationale is generalizable to a broad spectrum of adult neurological conditions marked by disrupted spinal circuitry. Pathological patterns of spinal map coactivation, synchronization, and topography alterations have been comprehensively documented in adult spinal cord injury, stroke, elderly, and hereditary spastic paraplegia integrity (Grasso et al., 2004; Monaco et al., 2010; Coscia et al., 2015; Martino et al., 2018; Dewolf et al., 2021), as well as for characterizing gait adaptation when using assistive exoskeletons (Zhvansky et al., 2022; Villani et al., 2024). The real-time nature of this framework makes it equally applicable for tracking neural degeneration or recovery in adult neurorehabilitation environments.
- Exploratory visual biofeedback: By transforming complex, high-density EMG signals into intuitive, smoothed spatiotemporal colormaps, the framework opens up new pathways for active biofeedback paradigms. Translating abstract muscle waveforms into real-time visual “hotspots” may allow patients to consciously perceive and attempt to modulate their motor output during gait. This volitional engagement could facilitate motor learning and the reinforcement of more physiological, segmentally differentiated activation patterns, particularly in populations where altered sensorimotor integration severely limits conventional gait correction.

### 4.2 Integration into clinical workflows and neuromodulation

One of the most promising applications of the proposed system is its ability to provide immediate, clinically meaningful feedback during gait assessment. A primary translational advantage of this real-time visualization tool is its potential integration directly into the workflow of physical therapists. During standard gait assessment or training sessions, therapists continuously apply manual facilitation, provide verbal cues, or modify assistive device configurations. Our system enables therapists to evaluate in real time how these moment-to-moment therapeutic interventions influence the recruitment and rostrocaudal separation of the patient’s lumbar and sacral motor pools. For example, the immediate visualization of a shift from pathological lumbosacral coactivation toward a more segregated and physiologically typical temporal structure may provide objective confirmation that a specific manual technique or postural adjustment is effectively engaging spinal locomotor networks.

The software accurately detected step onset and termination in real time using the leg oscillation angle, which is plotted alongside the spinal maps (Figures 3A,C). The system provides two complementary outputs: (1) a visual representation of lumbosacral motoneuron activity and (2) quantitative parameters characterizing motor pool activation within each stride (SMP1–SMP4). As illustrated in Figure 3, inter-stride variability was evident, reflecting natural fluctuations in muscle activity and locomotor output across steps. Nevertheless, despite this variability, consistent features of motor pool activation could be identified in the visualized spinal maps (Figures 3A,C). In TD children, spinal maps showed clear temporal segregation between lumbar and sacral segments, with lumbar segments predominantly active during early stance and sacral segments during late stance. This rostrocaudal differentiation was reflected by significant differences between SMP1 and SMP2 and by the absence of significant correlations between lumbar and sacral activation dynamics (SMP4 was not significantly different from zero; Figure 3B). In contrast, children with CP exhibited more synchronous activation of lumbar and sacral segments, evidenced by the absence of differences between SMP1 and SMP2 and by significant correlations between lumbar and sacral waveforms (Figures 3D, 4). Importantly, the differences captured by the spinal-map-based approach were evident even in children with CP who were able to walk independently and were classified as having mild motor impairment (GMFCS levels I–II, Table 1). This finding highlights the potential clinical utility of the system for detecting subtle alterations in spinal locomotor control.

In addition to therapist-oriented monitoring, the system may also support biofeedback applications, although these remain exploratory and require further validation, particularly in paediatric populations. By transforming complex EMG-derived spinal activity into intuitive visual representations, the system may allow patients to perceive and potentially modulate aspects of their motor output that would otherwise remain inaccessible. Such biofeedback could facilitate motor learning, enhance engagement during rehabilitation, and support the development of compensatory strategies or the reinforcement of more physiological activation patterns. This may be particularly relevant in individuals with CP, in whom altered sensorimotor integration often limits the capacity for volitional modulation of gait-related muscle activity.

Furthermore, real-time spinal maps of motor pool activation hold clear promise as a method for optimizing the neural control strategy and pattern of motor pool activation when children or adults walk in assistive exoskeletons (Zhvansky et al., 2022; Villani et al., 2024). Because an abnormal segregation of motor pool activity in spinal segments during prolonged usage of assistive technology may result in the development of abnormal plastic locomotor patterns or even irreversible changes in the spinal locomotor circuitry, a non-invasive, online assessment tool is highly desirable. Supplementing standard cinematic optimization with our near real-time performance measures would allow researchers and developers to better align an exoskeleton’s physical guidance forces with a patient’s active spinal pattern generation circuitry.

The system architecture is also highly compatible with integration into spinal neuromodulation paradigms, including transcutaneous and epidural electrical stimulation (Solopova et al., 2017; Wagner et al., 2018; Shapkova et al., 2020; Lorach et al., 2023). When configuring spinal stimulation parameters, clinicians are often constrained to static or predefined settings because of the difficulty of dynamically evaluating functional spinal cord activation during locomotion. The proposed framework could serve as an online monitoring tool, enabling immediate assessment of how changes in stimulation frequency, intensity, timing, or electrode geometry affect motor pool recruitment on a stride-by-stride basis. Such functionality could facilitate adaptive, closed-loop titration of stimulation parameters based on the immediate physiological response of the spinal cord with the characteristic activation hotspots during gait.

Previous work has already demonstrated the utility of spinal maps in neuromodulation settings. For example, Rowald and colleagues (2022) adapted the epidural stimulation parameters to the needs of individual patients with complete paraplegia and, in so doing, they achieved restoration of walking function using, among other measures, spatiotemporal spinal activity maps to characterize target hotspots. We propose that the use of real-time spinal maps may considerably facilitate the choice of stimulation parameters during locomotion stride by stride. This approach could support adaptive tuning of stimulation intensity, timing, and electrode configuration based on physiological responses rather than predefined settings. By providing a direct window into segmental motor pool dynamics during stimulation, the method may contribute to more precise neuromodulatory interventions and support the development of closed-loop control strategies.

The software could also be extended to incorporate bilateral maps of spinal motoneuron activation, enabling real-time monitoring of rostrocaudal shifts in the centre of bilateral motoneuron activity. Such shifts mirror variations in energetic demands associated with center-of-mass motion during gait in neurologically intact individuals (Cappellini et al., 2010). Notably, previous work has shown that providing centre-of-mass feedback can reduce the energetic cost of walking in individuals with hemiparesis (Massaad et al., 2010). Overall, the capacity to deliver immediate feedback, support biofeedback-based training, and integrate seamlessly with neuromodulation highlights the system’s potential not only as a diagnostic tool but also as a therapeutic and translational technology. These features reinforce its relevance for next-generation personalized gait rehabilitation paradigms.

To address clinical implementation barriers and the therapeutic viability of myoelectric interventions in paediatric neurorehabilitation, we previously conducted a comprehensive institutional survey and state-of-the-art review on the clinical relevance of surface EMG in cerebral palsy (Cappellini et al., 2020). While clinicians and physical therapists frequently acknowledge significant hurdles to widespread surface EMG usage due to demanding data-processing time constraints and complex signal interpretation pipelines, there remains a powerful consensus regarding its unique functional value in identifying pathological coordination mechanisms. Furthermore, contrary to traditional scepticism surrounding the efficacy of volitional pathway modulation in younger patient populations, numerous clinical trials have firmly established the safety, feasibility, and therapeutic efficacy of EMG driven biofeedback therapy in individuals with CP. Standard protocols utilizing immediate auditory and visual closed-loop feedback of peripheral muscle contractions have demonstrated documented success in reinforcing targeted muscle activation, suppressing antagonistic hyperreflexia, expanding active ranges of motion, and eliciting objective, long-term improvements in clinical gait speed and hand function coordination (Dursun et al., 2004; Bloom et al., 2010; Yoo et al., 2017; Booth et al., 2019). By upgrading standard, abstract single-channel muscle waveforms into an integrated, near real-time spatiotemporal topographic map of the underlying lumbosacral cord, our current user interface layout specifically aims to overcome the “difficult data interpretation” bottleneck cited by clinical staff, providing clinicians with an actionable visual toolkit to optimize these established biofeedback and motor learning paradigms directly at the bedside.

#### 4.2.1 Distinguishing current feasibility from prospective applications

Importantly, the present study must be interpreted strictly within its scope as a technical and methodological proof-of-concept and feasibility evaluation. The pilot validation was conducted on a small sample size consisting of eight children with mild cerebral palsy. While the stride-level statistics utilized herein successfully characterized within-subject variability, temporal consistency, and individual activation structure, they do not possess the statistical independence required for population-level or group-level inferential generalizations.

Consequently, while the system demonstrated high sensitivity in capturing individual, segment-specific abnormalities - such as the synchronous lumbosacral coactivation and localized lumbar hyper-intensity reflective of altered posture in the CP cohort (Figures 3, 4) - these differences serve as descriptive proof-of-concept examples rather than clinical validation of diagnostic utility. No formal clinical interventions, therapist workflow integrations, or closed-loop neuromodulation protocols were implemented in this work. These advanced interventional applications, alongside structured clinician usability testing and the direct deployment of patient biofeedback training, remain exploratory hypotheses and represent prospective research directions requiring rigorous, dedicated clinical trials before translational readiness can be claimed.

Nevertheless, the maps capture general features of motor pool activity (Figures 3, 4). The system is fully customisable and can be extended with additional algorithms and comparisons with the appropriate database of control subjects (given the effect of age, etc.), allowing adaptation to more complex experimental designs and future clinical applications.

#### 4.2.2 Limitations

Several limitations of our study merit consideration. First, although the number of recorded muscles during walking was limited to nine channels by default, previous work has shown that this subset effectively captures the major features of spinal maps (Ivanenko et al., 2013; La Scaleia et al., 2014) and represents a substantial portion of the lower-limb muscle cross-sectional area (Ward et al., 2009). For broader applications, the system is designed to be highly flexible, supporting the integration of up to 16 synchronized EMG channels.

An underlying physiological assumption of this methodology is that rectified surface EMG amplitude provides an indirect estimate of the net firing rate and population activity of the corresponding alpha-motoneuron pool (Yakovenko et al., 2002). Despite its simplicity, this assumption is generally well supported by classical electrophysiological models, as mean EMG amplitude scales nearly linearly with aggregate motor unit firing rate (Hoffer et al., 1987; Day and Hulliger, 2001). However, surface EMG recordings are inherently susceptible to cross-talk and movement artefacts, which cannot be fully eliminated in real time. Therefore, careful electrode placement and thorough pre-trial signal checks are essential operational prerequisites. Similarly, ensuring consistent visibility of motion-capture markers throughout each trial is critical, as severe marker occlusions can degrade stride phase segmentation and the subsequent temporal normalization of the spinal maps. In the present study, these procedures were rigorously followed at the start of each walking trial.

Second, the reconstructed spinal maps were derived from standardized, classical anatomical charts of segmental muscle innervation (Sharrard, 1964; Kendall et al., 2005), assigning identical motor pool distributions to all participants. Although some individual anatomical deviations have been reported (Phillips and Park, 1991; Stewart, 1992; Ranger et al., 2008; Van Schoor et al., 2015; Khasawneh, 2019; Nunès et al., 2023), available myotomal charts generally support this approach. Using standardised maps ensure methodological consistency and avoid the technical challenges of spinal fMRI, but also prevented us from capturing possible inter-individual variability in segmental organisation. Recent task-based spinal fMRI work suggests that functional “projectomes” may differ across individuals (Hernandez-Charpak et al., 2025), although current fMRI methods remain limited by motion sensitivity, small samples, uncertain test-retest reliability, and complex normalisation pipelines. Moreover, the tasks used in such studies (joint-specific movements, passive movements, muscle vibration) do not isolate individual muscles and largely engage somatosensory pathways, complicating interpretation of MN-specific activation. Given these constraints, non-invasive myotomal projections provide a practical, robust and reproducible framework that supports inter-subject comparisons and offers a relatively simple tool for the users (therapist and patient). Future integration of classical charts with emerging subject-specific functional mapping may improve precision, but current innervation maps (Kendall et al., 2005) appear sufficiently reliable for the spatial resolution achievable with EMG-based spinal mapping.

In sum, the proposed spinal mapping approach provides an indirect, model-based representation of segmental motor output. Its validity relies on two main assumptions: that rectified EMG amplitude is a proxy of motoneuron population activity, and that standardized myotomal charts adequately represent the rostrocaudal organization of motor pools. Consequently, spinal maps should be interpreted as functional approximations rather than direct measurements of spinal cord activity. Additional limitations include inter-individual anatomical variability and the inability to resolve interneuronal or supraspinal contributions to motor control.

Finally, the current implementation of the real-time system processes consecutive strides from a single body side at a time. This architectural constraint was deliberately selected to guarantee small computational latency and reliable visual rendering speeds during active streams. Although unilateral processing represents a limitation for the comprehensive evaluation of bilateral gait asymmetries - which are highly relevant in clinical hemiplegic or diplegic cohorts - the underlying data stream framework can be extended to parallel bilateral reconstruction in future software versions to yield insights into inter-limb coordination.

#### 4.2.3 Alternative approach

An alternative technical approach to our cycle-based system would be to generate completely continuous, frame-by-frame real-time maps that bypass gait event detection and time normalization entirely. Under such an architecture, each visual frame (map) would simply represent the instantaneous, raw projection of incoming EMG signals onto the spinal myotomal topography, with system lag limited only to the processing time and the monitor’s refresh rate. An example of this method (based on the recording of walking in one adult) is shown in Supplementary Video S2. This approach would have a different application that the present ones, it could be used to detect real time events, such as spinal events related to stumbling on an obstacle, or transient yield of the leg or foot drop once these images are displayed along with the quasi-synchronous video recording.

The cycle-based approach employed in this study and presented in Figures 1–4 differs from this continuous visualization. Here, each map is constructed after the completion of a gait cycle, allowing for a more interpretable characterization of the functional loci of MN activity within each stride (or after averaging multiple strides, Figure 2A). This cycle-based method also facilitates comparison with published data and enables computation of the performance indicators (SMP1–SMP4). An example of this stride-by-stride approach is shown in Supplementary Video S1.

## 5 Conclusion

We have presented a flexible, near real-time visualization platform that successfully maps multi-muscle surface electromyography onto the approximate rostrocaudal topography of lumbosacral motor pools on a stride-by-stride basis. While the system must currently be interpreted as a technical proof-of-concept tested on a small pilot sample, it demonstrates clear sensitivity in capturing distinct, individual features of neural coordination and structural motor pool synchronization (Figures 3, 4). The underlying software framework is highly customizable, permitting the adjustment of filtering parameters, myotomal interpolation charts, muscle selections, and advanced user-defined expansions. Ultimately, by providing immediate feedback on segmental motor output dynamics, this real-time methodology represents a promising translational tool for identifying patient-specific physiological markers and supporting next-generation paradigms in adaptive, personalized neurorehabilitation. The aim of this software is: activating and remodelling the spinal neuronal pathways to get closer to the typical evolutionary evolved neuromechanics of spinal locomotor circuitry functioning.

## Data Availability

The raw data supporting the conclusions of this article will be made available by the authors, without undue reservation.

## Supplementary material

SUPPLEMENTARY VIDEO S1 Example of near real-time, stride-by-stride spinal locomotor output visualization in a child with cerebral palsy (Participant ID: CP8). The top panel shows the participant during overground walking captured via the Vicon-Nexus system. The bottom panel shows the synchronized user interface screen displaying the reconstructed spinal map and associated spinal map parameters (SMP1-SMP4). The display is temporally synchronized such that the spatiotemporal map and quantitative parameters automatically compute and refresh at the exact termination of each consecutive stride (a total of 4 strides of the right leg are shown).

SUPPLEMENTARY VIDEO S2 Example of continuous real-time visualization of spinal locomotor output. This approach generates uninterrupted real-time maps that do not depend on gait event detection or time normalization. Each frame shows the instantaneous projection of EMG activity from the left (L) and right (R) legs of an adult participant onto the corresponding Sharrard (1955) motor pools illustrated on the left and Kendall’s myotoms in the middle panel.

## References

[B1] Abdelmohsen A. M. (2025). Artificial intelligence in biomechanics: a narrative review of current applications in diagnostic and physical rehabilitation. Physiother. Res. Int. 30, e70120. 10.1002/pri.70120 41137710

[B2] Allseits E. Lučarević J. Gailey R. Agrawal V. Gaunaurd I. Bennett C. (2017). The development and concurrent validity of a real-time algorithm for temporal gait analysis using inertial measurement units. J. Biomech. 55, 27–33. 10.1016/j.jbiomech.2017.02.016 28302315

[B3] Avaltroni P. Cappellini G. Sylos-Labini F. Ivanenko Y. Lacquaniti F. (2024). Spinal maps of motoneuron activity during human locomotion: neuromechanical considerations. Front. Physiol. 15, 1389436. 10.3389/fphys.2024.1389436 39108539 PMC11300930

[B4] Avaltroni P. Ivanenko Y. Villani M. Scordo G. Sylos-Labini F. Medici A. (2026). The role of independent walking in the maturation of the spinal locomotor output in children with cerebral palsy. Front. Physiol. 17, 1828195. 10.3389/fphys.2026.1828195 42212258 PMC13212187

[B5] Berens P. (2009). CircStat: a MATLAB toolbox for circular statistics. J. Stat. Softw. 31. 10.18637/jss.v031.i10

[B6] Bloom R. Przekop A. Sanger T. D. (2010). Prolonged electromyogram biofeedback improves upper extremity function in children with cerebral palsy. J. Child. Neurol. 25, 1480–1484. 10.1177/0883073810369704 20525944

[B7] Bohannon R. W. Smith M. B. (1987). Interrater reliability of a modified ashworth scale of muscle spasticity. Phys. Ther. 67, 206–207. 10.1093/ptj/67.2.206 3809245

[B8] Booth A. T. C. van der Krogt M. M. Harlaar J. Dominici N. Buizer A. I. (2019). Muscle synergies in response to biofeedback-driven gait adaptations in children with cerebral palsy. Front. Physiol. 10, 1208. 10.3389/fphys.2019.01208 31611807 PMC6776587

[B9] Capogrosso M. Wagner F. B. Gandar J. Moraud E. M. Wenger N. Milekovic T. (2018). Configuration of electrical spinal cord stimulation through real-time processing of gait kinematics. Nat. Protoc. 13, 2031–2061. 10.1038/s41596-018-0030-9 30190556

[B10] Cappellini G. Ivanenko Y. P. Dominici N. Poppele R. E. Lacquaniti F. (2010). Migration of motor pool activity in the spinal cord reflects body mechanics in human locomotion. J. Neurophysiol. 104, 3064–3073. 10.1152/jn.00318.2010 20881204

[B11] Cappellini G. Ivanenko Y. P. Martino G. MacLellan M. J. Sacco A. Morelli D. (2016). Immature spinal locomotor output in children with cerebral palsy. Front. Physiol. 7, 478. 10.3389/fphys.2016.00478 27826251 PMC5078720

[B12] Cappellini G. Sylos-Labini F. Assenza C. Libernini L. Morelli D. Lacquaniti F. (2020). Clinical relevance of state-of-the-art analysis of surface electromyography in cerebral palsy. Front. Neurol. 11, 583296. 10.3389/fneur.2020.583296 33362693 PMC7759523

[B13] Coscia M. Monaco V. Martelloni C. Rossi B. Chisari C. Micera S. (2015). Muscle synergies and spinal maps are sensitive to the asymmetry induced by a unilateral stroke. J. Neuroeng Rehabil. 12, 39. 10.1186/s12984-015-0031-7 25928264 PMC4411739

[B14] Day S. J. Hulliger M. (2001). Experimental simulation of cat electromyogram: evidence for algebraic summation of motor-unit action-potential trains. J. Neurophysiol. 86, 2144–2158. 10.1152/jn.2001.86.5.2144 11698507

[B15] Dewolf A. H. Sylos-Labini F. Cappellini G. Zhvansky D. Willems P. A. Ivanenko Y. (2021). Neuromuscular age-related adjustment of gait when moving upwards and downwards. Front. Hum. Neurosci. 15, 749366. 10.3389/fnhum.2021.749366 34744664 PMC8566537

[B16] Dominici N. Ivanenko Y. P. Lacquaniti F. (2007). Control of foot trajectory in walking toddlers: adaptation to load changes. J. Neurophysiol. 97, 2790–2801. 10.1152/jn.00262.2006 17251371

[B17] Dursun E. Dursun N. Alican D. (2004). Effects of biofeedback treatment on gait in children with cerebral palsy. Disabil. Rehabil. 26, 116–120. 10.1080/09638280310001629679 14668149

[B18] Dusane S. Shafer A. Ochs W. L. Cornwell T. Henderson H. Kim K.-Y. A. (2023). Control of center of mass motion during walking correlates with gait and balance in people with incomplete spinal cord injury. Front. Neurol. 14, 1146094. 10.3389/fneur.2023.1146094 37325225 PMC10262050

[B19] Grasso R. Ivanenko Y. P. Zago M. Molinari M. Scivoletto G. Castellano V. (2004). Distributed plasticity of locomotor pattern generators in spinal cord injured patients. Brain 127, 1019–1034. 10.1093/brain/awh115 14988161

[B20] Grillner S. El Manira A. (2015). The intrinsic operation of the networks that make Us locomote. Curr. Opin. Neurobiol. 31, 244–249. 10.1016/j.conb.2015.01.003 25599926

[B21] Hernandez-Charpak S. D. Kinany N. Ricchi I. Schlienger R. Mattera L. Martuzzi R. (2025). Towards personalized mapping through Lumbosacral spinal cord task fMRI. Imaging Neurosci. (Camb) 3, imag_a_00455. imag_a_00455. 10.1162/imag_a_00455 40800855 PMC12319805

[B22] Himmelmann K. Horber V. De La Cruz J. Horridge K. Mejaski-Bosnjak V. Hollody K. (2017). MRI classification system (MRICS) for children with cerebral palsy: development, reliability, and recommendations. Dev. Med. Child. Neurol. 59, 57–64. 10.1111/dmcn.13166 27325153

[B23] Hoffer J. A. Sugano N. Loeb G. E. Marks W. B. O’Donovan M. J. Pratt C. A. (1987). Cat hindlimb motoneurons during locomotion. II. Normal activity patterns. J. Neurophysiol. 57, 530–553. 10.1152/jn.1987.57.2.530 3559691

[B24] Ivanenko Y. P. Poppele R. E. Lacquaniti F. (2006). Spinal cord maps of spatiotemporal alpha-motoneuron activation in humans walking at different speeds. J. Neurophysiology 95, 602–618. 10.1152/jn.00767.2005 16282202

[B25] Ivanenko Y. P. Cappellini G. Poppele R. E. Lacquaniti F. (2008). Spatiotemporal organization of alpha-motoneuron activity in the human spinal cord during different gaits and gait transitions. Eur. J. Neurosci. 27, 3351–3368. 10.1111/j.1460-9568.2008.06289.x 18598271

[B26] Ivanenko Y. P. Dominici N. Cappellini G. Di Paolo A. Giannini C. Poppele R. E. (2013). Changes in the spinal segmental motor output for stepping during development from infant to adult. J. Neurosci. 33, 3025–3036a. 10.1523/JNEUROSCI.2722-12.2013 23407959 PMC6619203

[B27] Kendall F. McCreary E. Provance P. Rodgers M. Romani W. (2005). Muscles. Testing and Function with Posture and Pain. Baltimore: Lippincott Williams and Wilkins.

[B28] Khasawneh R. R. (2019). Influence of age, sex, height and lumber stenosis on the position of the Conus medullaris in adults. Int. J. Morphol. 37, 867–871. 10.4067/S0717-95022019000300867

[B29] Kinany N. Pirondini E. Micera S. Van De Ville D. (2023). Spinal cord fMRI: a new window into the central nervous system. Neuroscientist 29, 715–731. 10.1177/10738584221101827 35822665 PMC10623605

[B30] La Scaleia V. Ivanenko Y. P. Zelik K. E. Lacquaniti F. (2014). Spinal motor outputs during step-to-step transitions of diverse human gaits. Front. Hum. Neurosci. 8, 305. 10.3389/fnhum.2014.00305 24860484 PMC4030139

[B31] Lorach H. Galvez A. Spagnolo V. Martel F. Karakas S. Intering N. (2023). Walking naturally after spinal cord injury using a brain-spine interface. Nature 618, 126–133. 10.1038/s41586-023-06094-5 37225984 PMC10232367

[B32] Martini E. Fiumalbi T. Dell’Agnello F. Ivanić Z. Munih M. Vitiello N. (2020). Pressure-sensitive insoles for real-time gait-related applications. Sensors (Basel) 20, 1448. 10.3390/s20051448 32155828 PMC7085512

[B33] Martino G. Ivanenko Y. Serrao M. Ranavolo A. Draicchio F. Rinaldi M. (2018). Differential changes in the spinal segmental locomotor output in hereditary spastic paraplegia. Clin. Neurophysiol. 129, 516–525. 10.1016/j.clinph.2017.11.028 29353180

[B34] Massaad F. Lejeune T. M. Detrembleur C. (2010). Reducing the energy cost of hemiparetic gait using center of mass feedback: a pilot study. Neurorehabil. Neural Repair 24, 338–347. 10.1177/1545968309349927 19890020

[B35] Monaco V. Ghionzoli A. Micera S. (2010). Age-related modifications of muscle synergies and spinal cord activity during locomotion. J. Neurophysiol. 104, 2092–2102. 10.1152/jn.00525.2009 20685924

[B36] Nagano H. Said C. M. James L. Begg R. K. (2020). Feasibility of using foot-ground clearance biofeedback training in treadmill walking for post-stroke gait rehabilitation. Brain Sci. 10, 978. 10.3390/brainsci10120978 33322082 PMC7764443

[B37] Nierula B. Stephani T. Bailey E. Kaptan M. Pohle L.-M. G. Horn U. (2024). A multichannel electrophysiological approach to noninvasively and precisely record human spinal cord activity. PLoS Biol. 22, e3002828. 10.1371/journal.pbio.3002828 39480757 PMC11527246

[B38] Nunès A. Glaudot G. Lété A. Balci A. Lengelé B. Behets C. (2023). Measurements and morphometric landmarks of the human spinal cord: a cadaveric study. Clin. Anat. 36, 631–640. 10.1002/ca.24010 36647816

[B39] Phillips L. H. Park T. S. (1991). Electrophysiologic mapping of the segmental anatomy of the muscles of the lower extremity. Muscle Nerve 14, 1213–1218. 10.1002/mus.880141213 1766452

[B40] Ranger M. R. B. Irwin G. J. Bunbury K. M. Peutrell J. M. (2008). Changing body position alters the location of the spinal cord within the vertebral canal: a magnetic resonance imaging study. Br. J. Anaesth. 101, 804–809. 10.1093/bja/aen295 18936040

[B41] Rowald A. Komi S. Demesmaeker R. Baaklini E. Hernandez-Charpak S. D. Paoles E. (2022). Activity-dependent spinal cord neuromodulation rapidly restores trunk and leg motor functions after complete paralysis. Nat. Med. 28, 260–271. 10.1038/s41591-021-01663-5 35132264

[B42] Shapkova E. Y. Pismennaya E. V. Emelyannikov D. V. Ivanenko Y. (2020). Exoskeleton walk training in paralyzed individuals benefits from transcutaneous lumbar cord tonic electrical stimulation. Front. Neurosci. 14, 416. 10.3389/fnins.2020.00416 32528238 PMC7263322

[B43] Sharrard W. J. (1955). The distribution of the permanent paralysis in the lower limb in poliomyelitis; a clinical and pathological study. J. Bone Jt. Surg. Br. 37-B, 540–558. 10.1302/0301-620X.37B4.540 13271481

[B44] Sharrard W. J. (1964). The segmental innervation of the lower limb muscles in man. Ann. R. Coll. Surg. Engl. 35, 106–122. 14180405 PMC2311748

[B45] Solopova I. A. Sukhotina I. A. Zhvansky D. S. Ikoeva G. A. Vissarionov S. V. Baindurashvili A. G. (2017). Effects of spinal cord stimulation on motor functions in children with cerebral palsy. Neurosci. Lett. 639, 192–198. 10.1016/j.neulet.2017.01.003 28063935

[B46] Spedden M. E. O’Neill G. C. Tierney T. M. West T. O. Schmidt M. Mellor S. (2024). Towards non-invasive imaging through spinal-cord generated magnetic fields. Front. Med. Technol. 6, 1470970. 10.3389/fmedt.2024.1470970 39445170 PMC11496111

[B47] Stewart J. D. (1992). Electrophysiological mapping of the segmental anatomy of the muscles of the lower extremity. Muscle Nerve 15, 965–966. 1495514

[B48] Stroman P. W. Wheeler-Kingshott C. Bacon M. Schwab J. M. Bosma R. Brooks J. (2014). The current state-of-the-art of spinal cord imaging: methods. Neuroimage 84, 1070–1081. 10.1016/j.neuroimage.2013.04.124 23685159 PMC4371133

[B49] Sylos-Labini F. La Scaleia V. Cappellini G. Dewolf A. Fabiano A. Solopova I. A. (2022). Complexity of modular neuromuscular control increases and variability decreases during human locomotor development. Commun. Biol. 5, 1256. 10.1038/s42003-022-04225-8 36385628 PMC9669031

[B50] Tomlinson B. E. Irving D. (1977). The numbers of limb motor neurons in the human Lumbosacral cord throughout life. J. Neurol. Sci. 34, 213–219. 10.1016/0022-510x(77)90069-7 925710

[B51] Vacheva D. E. Drumev A. K. (2025). Kinesiological analysis using inertial sensor systems: methodological framework and clinical applications in pathological gait. Sensors (Basel) 25, 4435. 10.3390/s25144435 40732570 PMC12299135

[B52] Van Schoor A.-N. Bosman M. C. Bosenberg A. T. (2015). Descriptive study of the differences in the level of the Conus medullaris in four different age groups. Clin. Anat. 28, 638–644. 10.1002/ca.22505 25644516

[B53] Villani M. Avaltroni P. Scordo G. Rubeca D. Berger D. J. Kreynin P. (2024). Evaluation of EMG patterns in children during assisted walking in the exoskeleton. Front. Neurosci. 18, 1461323. 10.3389/fnins.2024.1461323 39513047 PMC11541598

[B54] Wagner F. B. Mignardot J.-B. Le Goff-Mignardot C. G. Demesmaeker R. Komi S. Capogrosso M. (2018). Targeted neurotechnology restores walking in humans with spinal cord injury. Nature 563, 65–71. 10.1038/s41586-018-0649-2 30382197

[B55] Ward S. R. Eng C. M. Smallwood L. H. Lieber R. L. (2009). Are current measurements of lower extremity muscle architecture accurate? Clin. Orthop. Relat. Res. 467, 1074–1082. 10.1007/s11999-008-0594-8 18972175 PMC2650051

[B56] Wenger N. Moraud E. M. Gandar J. Musienko P. Capogrosso M. Baud L. (2016). Spatiotemporal neuromodulation therapies engaging muscle synergies improve motor control after spinal cord injury. Nat. Med. 22, 138–145. 10.1038/nm.4025 26779815 PMC5061079

[B57] Yakovenko S. Mushahwar V. VanderHorst V. Holstege G. Prochazka A. (2002). Spatiotemporal activation of Lumbosacral motoneurons in the locomotor step cycle. J. Neurophysiology 87, 1542–1553. 10.1152/jn.00479.2001 11877525

[B58] Yang C.-C. Hsu Y.-L. Shih K.-S. Lu J.-M. (2011). Real-time gait cycle parameter recognition using a wearable accelerometry system. Sensors (Basel) 11, 7314–7326. 10.3390/s110807314 22164019 PMC3231731

[B59] Yoo J. W. Lee D. R. Cha Y. J. You S. H. (2017). Augmented effects of EMG biofeedback interfaced with virtual reality on neuromuscular control and movement coordination during reaching in children with cerebral palsy. NeuroRehabilitation 40, 175–185. 10.3233/NRE-161402 28222541

[B60] Zhao P. Alencastre-Miranda M. Shen Z. O’Neill C. Whiteman D. Gervas-Arruga J. (2024). Computer vision for gait assessment in cerebral palsy: metric learning and confidence estimation. IEEE Trans. Neural Syst. Rehabil. Eng. 32, 2336–2345. 10.1109/TNSRE.2024.3416159 38889045

[B61] Zhvansky D. S. Sylos-Labini F. Dewolf A. Cappellini G. d’Avella A. Lacquaniti F. (2022). Evaluation of spatiotemporal patterns of the spinal muscle coordination output during walking in the exoskeleton. Sensors (Basel) 22, 5708. 10.3390/s22155708 35957264 PMC9370895

